# Finite Element Analysis of Different Osseocartilaginous Reconstruction Techniques in Animal Model Knees

**DOI:** 10.3390/ma16072546

**Published:** 2023-03-23

**Authors:** Cosmin Cosma, Dragos Apostu, Cristian Vilau, Alexandru Popan, Daniel Oltean-Dan, Nicolae Balc, Gheorghe Tomoaie, Horea Benea

**Affiliations:** 1 Department of Manufacturing Engineering, Technical University of Cluj-Napoca, 400641 Cluj-Napoca, Romania; cosmin.cosma@tcm.utcluj.ro (C.C.);; 2 Department of Orthopedics and Traumatology, Iuliu Haţieganu University of Medicine and Pharmacy, 400132 Cluj-Napoca, Romania; 3 Department of Material Resistance, Technical University of Cluj-Napoca, 400641 Cluj-Napoca, Romania; 4 Academy of Romanian Scientists, 050044 Bucharest, Romania

**Keywords:** finite element analysis, cartilage lesion, bone marrow concentrate (BMC), adipose-derived stem cells concentrate (ASC), porous collagen implant

## Abstract

Lesions of the articular cartilage are frequent in all age populations and lead to functional impairment. Multiple surgical techniques have failed to provide an effective method for cartilage repair. The aim of our research was to evaluate the effect of two different compression forces on three types of cartilage repair using finite element analysis (FEA). Initially, an in vivo study was performed on sheep. The in vivo study was prepared as following: Case 0—control group, without cartilage lesion; Case 1—cartilage lesion treated with macro-porous collagen implants; Case 2—cartilage lesion treated with collagen implants impregnated with bone marrow concentrate (BMC); Case 3—cartilage lesion treated with collagen implants impregnated with adipose-derived stem cells (ASC). Using the computed tomography (CT) data, virtual femur-cartilage-tibia joints were created for each Case. The study showed better results in bone changes when using porous collagen implants impregnated with BMC or ASC stem cells for the treatment of osseocartilaginous defects compared with untreated macro-porous implant. After 7 months postoperative, the presence of un-resorbed collagen influences the von Mises stress distribution, total deformation, and displacement on the *Z* axis. The BMC treatment was superior to ASC cells in bone tissue morphology, resembling the biomechanics of the control group in all FEA simulations.

## 1. Introduction

Articular cartilage serves as a support and load transmitter between bones, providing a low friction bearing surface [1]. Lesion of the articular cartilage is a frequent pathology that leads to pain and functional impairment, being caused by acute traumatic lesions, chronic degenerative alterations, repetitive micro-trauma, developmental articular pathologies, or metabolic factors [2]. Researchers determined that the minimum mechanical impact that can cause cartilage micro-cracking is between 5 to 20 MPa [3,4,5]. The articular cartilage lesion has an impaired healing capacity due to the low number of specialized cells, lack of blood vessels, and lack of undifferentiated stem cells.

Multiple complex surgical techniques have been studied for the treatment of articular cartilage lesions, but none of them proved to be effective in repairing cartilage lesions with a tissue closely resembling the native cartilage in terms of structure and function [6,7,8,9]. As a result, numerous in vitro and in vivo research have tried to further study the physiopathology of focal cartilage lesions and their repair to propose a better treatment [10,11,12]. Due to intra-operative difficulties, high costs, and ethical issues regarding chondrocyte culture and implantation, methods based on mesenchymal stem cells and implants are under development as key elements for cartilage repair or regeneration [9,13].

The main methods used for autologous mesenchymal stem cell harvesting are represented by bone marrow concentrate (BMC) and adipose-derived stem cells (ASC) from the stromal-vascular fraction of adipose tissue concentrate (SVF). Although the cells following both techniques share many characteristics, some differences in immunophenotypic differentiation potential and immunomodulatory activities do exist [14]. Currently, insufficient data exists on subchondral and trabecular bone tissue behavior after these types of cartilage treatments.

To enhance the results of medical intervention, imagistic examinations such as computed tomography (CT) and magnetic resonance imaging (MRI) are used for preoperative planning. These 2D data can be used to virtually re-create knee structures within the region of interest, and thus finite element analysis (FEA) can be developed. The FEA studies can precisely simulate and evaluate the articular aspect and biomechanics throughout normal post-operative activities and evaluate the tension distribution in knee structures while limiting the number of specimens needed for statistical analysis [11,15,16,17,18,19].

The aim of the present study was to evaluate the effect of two different compression pressures applied to three types of cartilage repair techniques using FEA methods on sheep knee models. The three osseocartilaginous reconstruction techniques used were: (1) collagen type I/III porous implant, (2) collagen type I/III porous implant impregnated with BMC cells, and (3) collagen type I/III porous implant infused with ASC. The first compression pressure tested was 0.38 MPa, representing the normal value for a sheep’s knee during gaiting. The second compression pressure was 0.76 MPa, and it simulates a post-operative traumatic injury following a fall or a direct impact. These FEA simulations were developed, including the thermal conditions of the knee joint. To the best of our knowledge, this is the first study where the biomechanical behavior of osseocartilaginous reconstruction using collagen implants and mesenchymal stem cells is investigated using an FEA method.

## 2. Materials and Methods

### 2.1. Animal Model

We performed the in vivo study on Turcan breed female sheep with no clinical health problems. This study was conducted according to the European Union directive number 63/2010 and Romanian law number 43/2014 and was approved by the Ethics Commission of the University of Medicine and Pharmacy “Iuliu Hatieganu” Cluj-Napoca, Romania (no. 237/19 June 2014). The mean age of ovine subjects was 2.5 ± 0.1 years, and their body weight was 50 ± 2 kg. Preoperative preparation consisted of 14 days of quarantine, 24 h of fasting, and local shaving. The animals were divided into four Cases (three repair types + control), each animal being treated as described below.

### 2.2. Surgical Procedure

**Figure 1:**
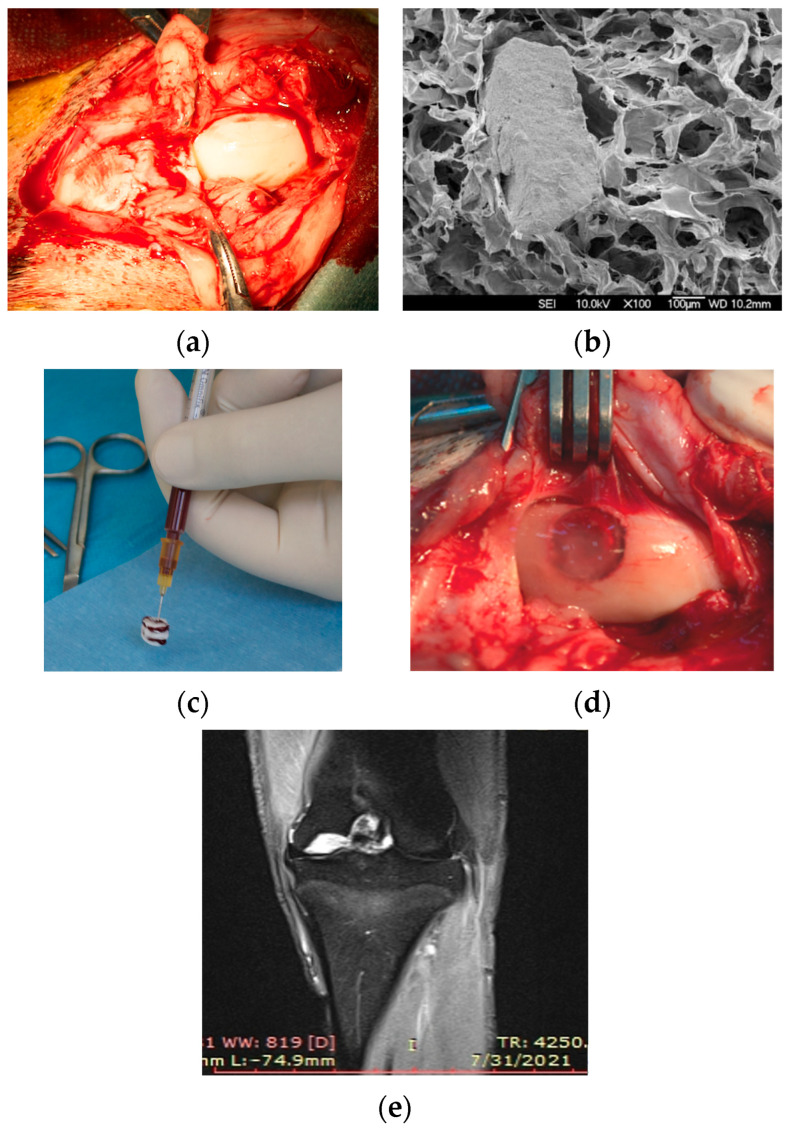
(**a**) Medial parapatellar approach over the left knee; (**b**) SEM image of porous collagen graft containing hydroxyapatite and tricalcium phosphate; (**c**) Injection of stem cells concentrate into the porous collagen implant; (**d**) Implant fixation in the defect using fibrin sealant; (**e**) MRI image after surgical intervention.

The surgical interventions were performed under general anesthesia and in aseptic conditions. After skin incision and subcutaneous dissection, a lateral patellar dislocation was performed, revealing the medial femoral condyle of the left knee (Figure 1a). The osteochondral defect was made using a drill with an 8 mm diameter, at a depth of 4–5 mm, or until a local haemorrhage was achieved (grade IV Outerbridge osteochondral defect).

An 8 mm diameter cylindrical collagen I/III implant (MatriBone Ortho^®^, Biom’Up, Saint-Priest, France) was applied to fill the articular defects (Figure 1b). This porous implant is a biphasic bone substitute with a resorbable collagen matrix containing a dispersion of hydroxyapatite (HA) and tricalcium phosphate (β-TCP). In Case 1, the treatment contains just the collagen porous implant. The same type of collagen implants was used as scaffolds for mesenchymal stem cells. The preparation of stem cell concentrates from the iliac crest and from the adipose tissue was done in the same surgical procedure using the dedicated kit Concemo^®^ (Proteal Soluciones Bioregenerativas, S.L., Barcelona, Spain), implying the harvesting of aspirates and centrifugation with separation of BMC or SVF. These concentrates were tested by flow cytometry for the presence and concentration of mesenchymal stem cells CD44. The platelet rich plasma (1 mL) was obtained during the same intervention from 20 mL. of circulating blood, using the kit OrthoPras^®^ (Proteal Soluciones Bioregenerativas, S.L., Barcelona, Spain). Figure 1c shows how the porous collagen implants were injected with 1 mL of BMC (Case 2), respectively, ASC mixed with platelet rich plasma (Case 3). All the implants were sealed in place with autologous fibrin glue (Tisseel Lyo^®^; Baxter, Deerfield, IL, USA). Implant fixation is presented in Figure 1d,e. Incision suture and dressing were performed afterwards.

Postoperatively, the sheep were placed in a closed environment with water and food ad libitum. Euthanasia was performed at 7 months following a surgical procedure using an overdose of pentobarbital (100 mg/mL) and after the collection of blood samples. The knee samples were prepared for CT investigations as described below.

### 2.3. CT Scanning and 3D Modelling

Cone beam computed tomography (CT) images at knee level were obtained using a Siemens scanner (Scope^®^, Erlangen, Germany) and Syngo CT VC28 software. CT scanning was performed preoperatively and 7 months postoperatively. The following scan parameters were configured: 130 kV, 7 mA, maximum 200 slices, 0.14 mm layer thickness, a 13.5 cm × 22.5 cm field of view, and an exposure time of 12 s. The CT images were imported into the MIMICS (Materialise^®^, Leuven, Belgium) and InVesalius (CTI^®^, Amarais—Campinas, Brazil) software’s, where 3D masks of tibia and femur bone tissues were obtained. To mimic bone as closely as possible, the tissue areas were segmented manually or by combining some threshold and morphological operations for segmentation of binary images [17,20,21,22,23]. It was demonstrated that the subchondral bone alterations are emerging as considerable clinical problems associated with articular cartilage repair [2]. For this reason, the virtual model of subchondral tissue was introduced.

**Figure 2:**
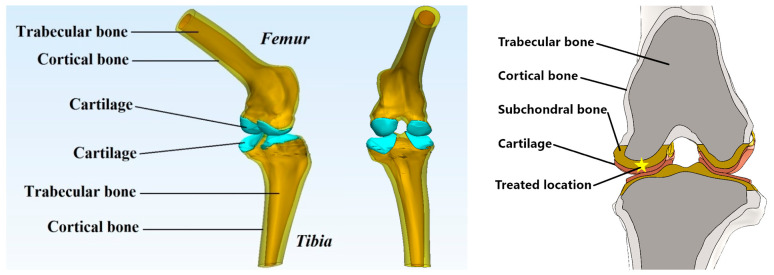
Virtual reconstruction of bone tissues, the designed cartilage, and treated location of Case 0 (Healthy); The yellow asterisk indicates where the collagen implant was inserted.

To avoid the anatomical-related differences, the present study was undertaken with the same virtual knee tissues (Figure 2); just the treated area was edited depending on the osseocartilaginous reconstruction appearance. To reproduce accurately the treated zones, a close examination of CT images was done, searching for the dimensions and localizations of un-resorbed collagen (UC) at 7 months postoperatively.

Using Creo Parametric software (Parametric Technology Corporation, Boston, MA, USA), we designed the virtual cartilage according to literature recommendations regarding the ovine knee joint [24,25]. The femur cartilage was designed with 0.9–1.1 mm thickness and the tibial cartilage with 1.1–1.3 mm. Moreover, the synovial fluid within the joint was also represented with 0.03 mm thickness [1,26]. Finally, three virtual femur-cartilage-tibia joints were created at real scale for each treatment applied (Case 1, Case 2, and Case 3) and a healthy one for control (Case 0—Figure 2). Considering the literature recommendations regarding the geometrical concentrators of tension and the methods of eliminating them [17,27], we opted for geometry parameterization of all the virtual models within the femur-cartilage-tibia joint via computer-aided design methods.

### 2.4. Knee Tissues Properties

The FEA study was undertaken using physical and mechanical characteristics of bone structures from various sources, focused on determining the bone tissues elasticity in large animals (including ovine). In Table 1, the Young modulus, Poisson ratio, density, compressive strength, and thermal properties of bone tissues, cartilage tissues, and synovial fluid are detailed. These characteristics have been set up, and we opted for homogeneous, isotropic, and elastic linear behavior. The synovial fluid within the joint was considered a waterproof membrane.

Based on our previous postoperative histological evaluation [28], in non-regenerated defects were found especially micro-fibrils of collagen. For this reason, we consider that this UC possesses 5000 MPa modulus of elasticity. This Young modulus value of collagen fibrils was determined in rat tail or tendons, respectively, in bovine Achilles tendons [29,30]. The triple helix tertiary structure of collagen fibrils gives the high tensile strength, great flexibility, and it can be mineralized [31,32]. Table 1 details the physical-mechanical and thermal characteristics of knee tissues used in the present FEA study.

**Table 1:** Physical-mechanical and thermal characteristics of knee tissues used in FEA study (mean values).

Tissue	Young Modulus [MPa]	Poisson Ratio	Density [g/cm^3^]	Compressive Strength [MPa]	Specific Heat [10^3^ J/kg °C]	Thermal Expansion Coefficient [10^−6^/°C]	Thermal Conductivity [J/mm * s * °C]	References
Trabecular bone	1500	0.30	0.61	2–16	0.44	10	0.58	[1,33,34,35,36,37]
Cortical bone	16,160	0.33	2.45	100–147				[1,34,35,36,37,38,39]
Subchondral bone	19,800	0.30	1.79	64				[10,36,37,40,41]
Cartilage	0.8	0.40	1.1	5–20 ^#^	0.32	N/A	0.21	[1,27,42,43,44,45]
UC	5000	0.30	1.32	5–100 ^##^	1.6	5	0.57	[5,29,30,31,46,47,48,49]
Synovial fluid	1	0.49	1	-	0.39	3.9 *	0.62	[1,26,45]

^#^ Tensions that cause lesions in cartilage [50]; ^##^ Starting from 5 MPa local stress, the collagen fibril can initiate and develop the degeneration process [5]; * Like blood plasma at 35–40 °C.

### 2.5. Conditions of FEA Simulations

The 3D models of the knee joint were uploaded into the ANSYS software, where FEA simulations were developed. The sensitivity of the FEA model to mesh size was tested by applying different meshes with decreasing element sizes down to 0.5 mm. The meshing process resulted in approximately 390,000 elements and 680,000 nodes.

**Figure 3:**
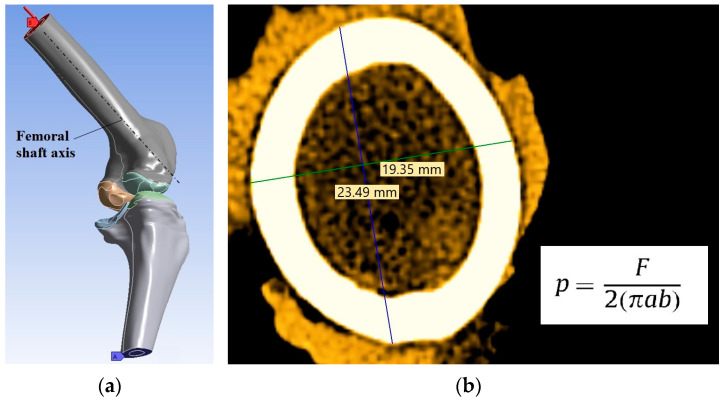
(**a**) FEA conditions: A—Constraint zones (blue area), B—Pressure zone and its direction (red area); (**b**) Femur cross-section and equation used to calculate the pressure.

The effect of each treatment on the mechanical response of bone tissue was simulated under compression conditions typical of the stance phase of gait, maintaining the treated area in contact with the opposite cartilage (see Figure 3a). The bottom plane the of tibia was rigidly constrained in all directions, and on the top plane of the femur was applied the compression pressure (Figure 3a, blue area). Since the permeability of cartilage is greater in the direction parallel rather than orthogonal to the orientation of the UC, free fluid flow was allowed only through the inner surfaces of reconstruction but was restricted elsewhere [17].

The knee joint force was applied on the bottom plane of the femur as a pressure (*p*) calculated with the equation detailed in Figure 3b, where *F* is the normal force on the whole knee joint, *a* and *b* are the major and minor axes lengths of the elliptical cross section of the femur. The force acting on the sheep’s knee joint is 2.1–2.25 times the weight of the animal [1,51,52]. Considering these recommendations and the fact that the mean animal weight was 50 kg, a pressure of 0.38 MPa was calculated utilizing femoral dimensions and the equation presented in Figure 3b. This pressure was applied to the virtual knee, and it represents a common situation in joints during daily activities. Moreover, a pressure of 0.76 MPa was applied. This high pressure was introduced to analyze the knee behavior after a traumatic injury at osseocartilaginous reconstruction level (for example falls or direct blows of the knee which was surgically treated 7 months ago). Every pressure was applied constantly and uniformly, and the direction of it was parallel with the axis of femoral diaphysis, as mentioned in Figure 3a.

Knowing the possibility of articular cartilage destruction by a mechanical-biological pathway at various levels of temperature, we established for each 3D tissue the following thermal conditions: the cartilages have a 31.4 °C temperature (measured at the intra-articular level) and 34.8 °C for the other bone tissues [28,45,53,54,55]. Using these FEA conditions and applying for each tissue the corresponding characteristics (detailed in Table 1), the biomechanical behavior was investigated.

## 3. Results

### 3.1. Evaluation of Bone Changes in Animal Model

**Figure 4:**
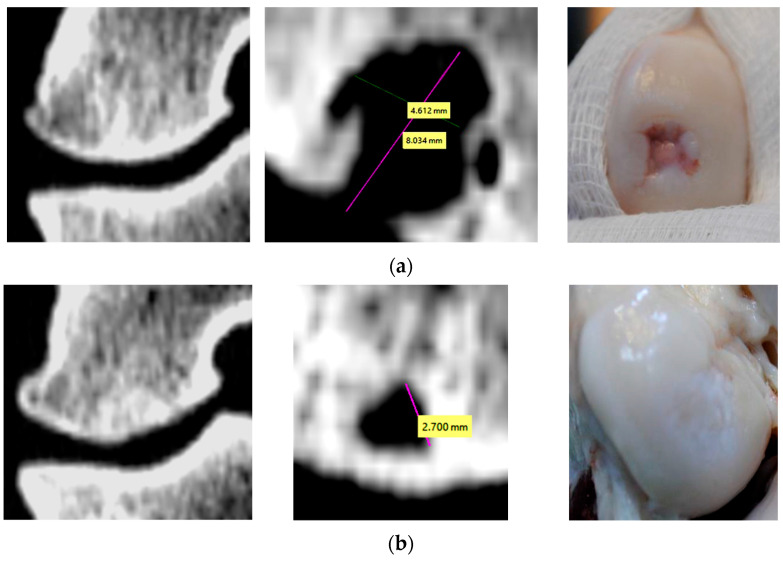

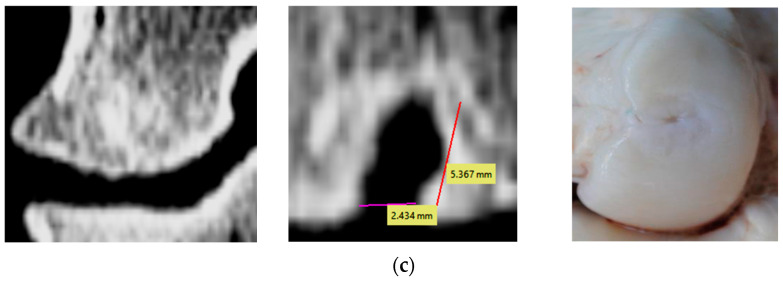
Comparative post-operative CT images of surgical treated areas (coronal and sagittal views): (**a**) Case 1 (just porous collagen); (**b**) Case 2 (Collagen + BMC); (**c**) Case 3 (Collagen + ASC).

The CT images representing the maximum dimensions of the non-regenerated zones at 7 months postoperatively in all treatment cases are shown in Figure 4. Moreover, coronal views show the new bone tissue that has regenerated in the lateral walls of the reconstructions. The density of these new bone structures from the lateral wall was measured, resulting in values between 1840 and 2100 Hounsfield units. This interval corresponds to adult bone tissue. Additionally, the homogeneity of these bone structures regenerated on defect walls is demonstrated by a low deviation of Hounsfield units, regardless of the surgical treatment applied. In all treatment Cases, the bone grew from the periphery to the center of the lesion, but the apex of the defect was not filled completely with trabecular bone (Figure 4).

### 3.2. Stress Distribution

**Figure 5:**
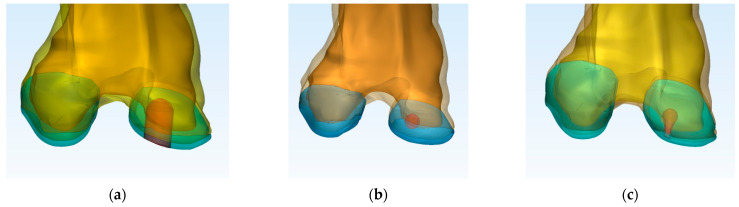
Location and the shape of UC after 7 months postoperatively: (**a**) Case 1 (just porous Collagen); (**b**) Case 2 (Collagen + BMC); (**c**) Case 3 (Collagen + ASC).

The virtual knee model of each treatment is presented in Figure 5. The shape and location of the UC at 7 months follow-up could be seen. To eliminate the geometrical concentrators of tension during FEA investigation, the anatomical shape of UC was limited (e.g., the virtual UC is a sphere for Case 2; Figure 5b).

**Figure 6:**
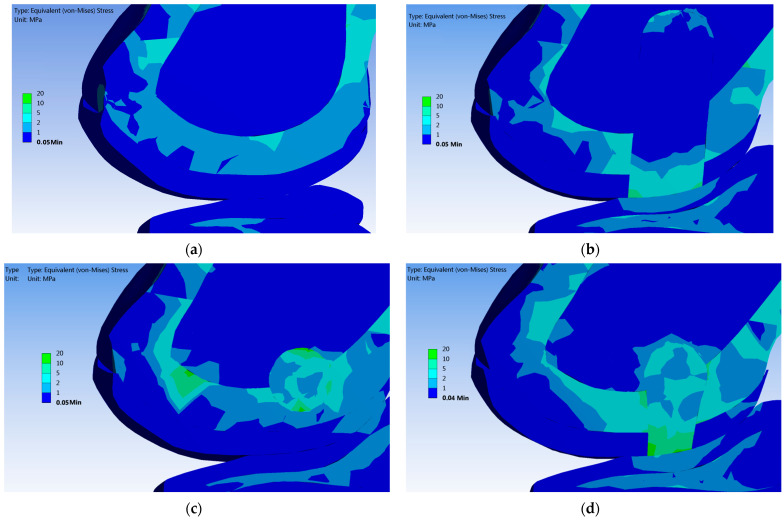
Distribution of von Mises stress in contact area of cartilage, after applying a normal pressure of 0.38 MPa: (**a**) Case 0 (healthy knee); (**b**) Case 1 (just porous Collagen); (**c**) Case 2 (Collagen + BMC); (**d**) Case 3 (Collagen + ASC).

Figure 6 shows the FEA results of static simulation in each case, examined after the normal knee pressure was applied. Von Mises tension distribution within the tissues is shown in cross-sections. These images represent the tensions within knee structures, which were calculated in the most representative areas after a compression pressure of 0.38 MPa was applied (Figure 6). The stress distribution is similar even after a 0.76 MPa compression pressure is applied. The difference is given by the intensities of tensions in each tissue. To summarize all the results obtained, Table 2 was elaborated. Here, the maximum tensions calculated are presented. Moreover, the limit of compression strength is mentioned for every tissue.

After it was applied the normal pressure (0.38 MPa), the UC conducted tensions up to 2 MPa in the tibial cartilage and femoral trabecular bone (Figure 6b–d). On the contact surfaces between the UC and the femoral cartilage, there are tensions between 5 to 15 MPa (Figure 6b–d). We observed that, if the femoral subchondral bone is not completely regenerated, this tissue accumulates higher tensions up to 11 MPa compared with a healthy knee. These aspects are noted in Table 2, and they can be seen in Figure 6.

Applying a constant compression pressure of 0.76 MPa, the UC is loaded with tensions up to 21 MPa, and they are distributed in femoral trabecular bone and tibial cartilage (Table 2). Thus, the apex of the defect distributes high tensions in the femoral trabecular bone, which are up to 5 MPa (see Case 1 and Case 3 in Table 2). In tibial trabecular bone, the tensions are under 2 MPa in every simulation.

### 3.3. Deformation and Displacement in Reconstruction Region

**Figure 7:**
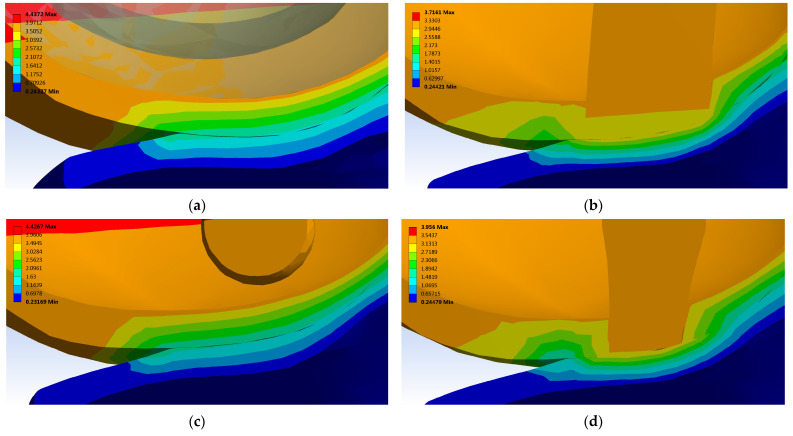
Total deformation in contact area of cartilage after applying a normal pressure of 0.38 MPa: (**a**) Case 0 (healthy knee); (**b**) Case 1 (just porous Collagen); (**c**) Case 2 (Collagen + BMC); (**d**) Case 3 (Collagen + ASC).

Figure 7 illustrates the total deformations in contact areas where the treatments have been applied. Following a pressure of 0.38 MPa, the healthy contact areas of tibial cartilage have a total deformation of maximum 1.8 mm (Figure 7a). In Case 1 and Case 3, the tibial cartilage undergoes a higher deformation of 2.6–2.7 mm (Figure 7b,d).

A reduced deformation similar to the ones determined in healthy cartilage was in Case 2. Here the total deformation of cartilage is 2.1 mm (Figure 7c). Increasing the pressure to 0.78 MPa has multiplied by two times the total deformations in Case 1 and Case 3, compared with normal one (0.38 MPa pressure).

**Figure 8:**
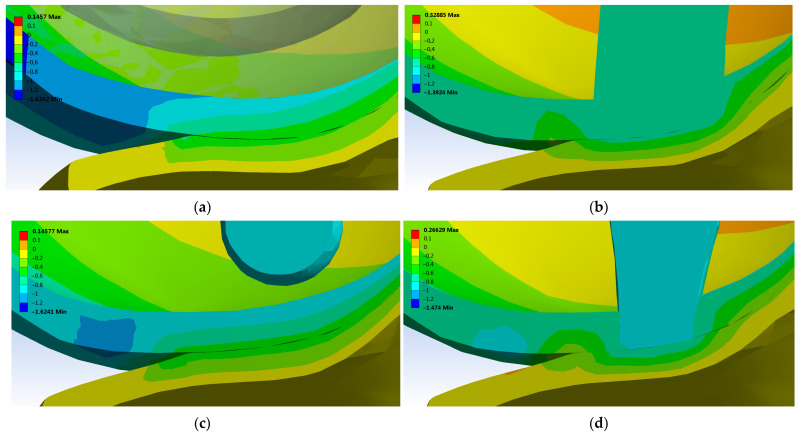
Directional deformation in *Z* axis of femoral and tibial cartilage after applying a normal pressure of 0.38 MPa: (**a**) Case 0 (healthy knee); (**b**) Case 1 (just porous Collagen); (**c**) Case 2 (Collagen + BMC); (**d**) Case 3 (Collagen + ASC).

The displacement was investigated in the loading direction for both tibial and femoral cartilage (*Z* axis) and the results are detailed in Figure 8. The healthy ovine knee presents a displacement between −1 mm to −0.4 mm for femoral cartilage and between −0.4 mm to 0 mm for tibial cartilage (Case 0, Figure 8a). Similar displacement behavior was obtained in Case 2 (Figure 8c). In reconstruction areas, the displacement of tibial cartilage was −0.7 mm in Cases 1 and Case 3. When doubling the compression pressure, in Cases 1 and Case 3, the femoral cartilage displacement was between −1.5 and −0.4 mm, while tibia cartilage displacement was between −1.4 and −0.1 mm. In this scenario, the reconstruction with UC influences the displacement of the cartilage compared with a healthy knee and the displacement on the *Z* axis in terms of the femoral cartilage was down to −2 mm and in case of tibia cartilage was −0.9 mm.

## 4. Discussion

Currently, the necessity of cells being used for chondral lesions’ repair is under debate worldwide, and the scientific literature does not yet provide an answer for this question [13,56,57,58,59]. Another issue is related to the efficiency of different types of cells that can be applied, such as chondrocytes or mesenchymal stem cells derived from adipose tissue, bone marrow, the bloodstream, synovial membrane, and even the umbilical cord. This in vivo research proves that the new type of macro-porous collagen I/III implant enhanced with multipotent mesenchymal cells contributes to intensification of osteochondral and trabecular regeneration 7 months postoperatively. It is also compatible and increases local repair without important adverse reactions. Compared with the classic treatment (with collagen and micro-fractures for local stimulation of mesenchymal cells from bone marrow), it was observed that the enhancement of porous collagen implants with BMC or ASC stimulates cartilage development.

### 4.1. Cartilage Tissue

Following the treatment just with porous collagen (Case 1), after 7 months postoperatively, the cartilage tissue was developed mostly on the lateral walls of the defect. The regenerated surfaces contain thin and irregular cartilage with obvious defects and cracks surrounding them, aspects related also by other studies [60,61,62]. From a cartilage regeneration perspective, the present study shows limited differences between BMC treatment and ASC, with this tissue being significantly reconstructed.

Because cartilage structure is very elastic, it distributed the tension from the femur to the tibia near the reconstructed defect. Applying a normal compression force, the contact pressure of a healthy joint was up to 1 MPa and was 2 times higher in each treated knee joint. Similar values of contact pressure in healthy knees treated with a double-curved implant made of metal was determined in another study [1]. The contact pressure increases in Case 1 and Case 3 because the tibial cartilage is in direct contact with the UC. Even if the knee joint is loaded with a trauma force, the tension from cartilages is maximum 4 MPa (see Table 2).

A reduced deformation, such as the healthy cartilage, was determinate in Case 2 because the femoral cartilage is completely regenerated (Figure 7). Likewise, if there is no direct contact between the UC and tibial cartilage, the displacement on the *Z* axis is similar to that of a healthy knee (Figure 8, Case 2). Increasing the compression pressure to 0.78 MPa leads to double the total deformation and displacement on the *Z* axis in the femoral and tibial cartilages. In general, the displacement in loading direction had values appropriate to other studies focused on tibio-femoral joints [61]. Using special micro-devices, further in vivo investigation of displacement after osseocartilaginous reconstruction could be developed [62,63,64,65].

### 4.2. Un-resorbed Collagen

In all the treatments applied, the postoperative examinations revealed that the bone gaps presented in the CT images (Figure 4) are made of fibro-cartilage tissue (mainly with collagen type I) [28]. Initiation and development of the collagen fibril degradation can occur locally, in regions where the stress exceeds the limit of 5 MPa. This threshold was determined experientially (data from the Osteoarthritis Initiative [5]). Actual investigation shows some areas of reconstruction that contain UC (consist of collagen micro-fibril), which possess tensions between 7 to 15 MPa after the normal compression pressure was applied (Figure 6b,d). This fact could be a possible explanation of why the cartilage plate did not completely regenerate after 7 months postoperatively, especially in Case 1.

The presence of UC in different areas influences considerably the results of von Mises stress distribution, total deformation, and displacement on the *Z* axis. Applying a pressure of 0.38 MPa, the contact area of healthy tibial cartilage has a deformation of maximum 1.8 mm (Figure 7a). In Case 1 and Case 3, where there is direct contact between the UC and tibial cartilage, the tibial cartilage is subject to a higher total deformation and displacement on the *Z* axis (Figure 7b,d).

### 4.3. Subchondral and Trabecular Bones

Based on descriptive analysis and identification of intralesional osteophyte [2], we observed that the subchondral bone has the tendency to be repaired after application of treatments in Cases 2 and Case 3. The dimension of the defect is significantly reduced, and in Case 2, the subchondral bone is nearly completely regenerated after 7 months. On the other hand, in Case 1 we detected a residual bone hole in subchondral plate and a retraction process of trabecular bone deep into the defect apex after 7 months postoperatively (with formation of pseudo-cysts, Figure 4a).

Because the subchondral bone is often involved in defects of the articular cartilage that are difficult to treat, it is associated with different articular cartilage repair techniques [2]. The current FEA research suggests that if the subchondral bone plate is not fully regenerated, the stress distribution could be conducted in the trabecular bone, especially at the apex of the reconstructed defect. Therefore, a slow development of subchondral bone leads to a gradual retraction of the trabecular bone. This phenomenon was observed in Case 1 (Figure 4). In Case 2, the subchondral bone is almost entirely repaired at 7 months, and it distributes reduced values of tension in the trabecular bone. A tailored algorithm based on continuous CT analysis of subchondral bone changes demonstrated similar results for a specific postoperative distinction between intra-lesional osteophytes, residual microfracture holes, peri-hole bone resorption, and subchondral cyst formation [2].

After the present treatments, the trabecular bone has not fully regenerated. The BMC and ASC treatments activated trabecular regeneration predominantly in the lateral walls of the defects. If a normal force is applied, the trabecular tissue of the femur possesses 2 MPa tension. Because collagen type I (or fibrils) within the UC reconstruction is more rigid than the trabecular bone surrounding it, it allows insertion of 2 MPa tensions, a value that corresponds to the cancellous bone compressive strength limit [35]. This fact could contribute to trabecular bone retraction from the defect region (Figure 4, Case 1, and Case 3), noting that the highest tensions are always localized in the apex defect region (Figure 6).

The actual porous implant is a biphasic bone substitute with a resorbable collagen matrix containing a dispersion of HA and β-TCP. In the bioceramics field, more than 400 forms, compositions, and trademarks are currently either in use or under consideration in many areas of orthopedics [66,67]. Positive results were reported, but insufficient data exists on subchondral and trabecular bone tissue behavior after these types of cartilage treatments.

There are some limitations in the present study that should be considered. Firstly, the virtual model of the knee was modified just in the osseocartilaginous reconstruction to avoid the anatomical-related differences. Secondly, the effect of cartilage repair was limited to 7 months. However, the un-resorbed collagen was investigated, and it was found to be mainly collagen type I, which is in the fibro-cartilage tissue. These pilot FEA results show that the tension distribution in the knee joint could be similar to a healthy one if the cartilage defect is treated with a collagen implant impregnated with BMC stem cells after 7 months post-operatory (Case 0 vs. Case 2).

Rapid developments in the field of materials and production technologies have made it possible to produce new types of complex porous implants [63,66,67]. To transfer these technologies to clinical practice, material science and tissue engineering need to be closely assisted by biomedical researchers to confer the safety risk assessment and efficacy at high standards [68,69,70]. The results obtained in this research contribute to the understanding and development of future treatments focused on osteochondral lesions and cartilage repair. However, extensive studies are needed to evaluate the application of these pilot treatments on an animal model or even in clinical trials to identify better treatment methods that have a higher reproducibility, lower morbidity, and risk. Future research work is required to examine the effect of osteocartilaginous reconstructions treated with porous collagen and stem cells under dynamic loading situations.

## 5. Conclusions

This pilot study showed better results in bone changes when using porous collagen implants impregnated with stem cells for the treatment of osteocartilaginous defects. The presence of UC in the treated area influences significantly the von Mises stress distribution, total deformation, and displacement on the *Z* axis. The BMC treatment was superior to the ASC treatment in cases of bone tissue morphology, resembling the biomechanics of the control group in all finite analysis simulations.

## Figures and Tables

**Table 2:** Maximum von Mises stress in contact region after applying a normal pressure vs. a high pressure.

Knee Tissue	Compression Pressure Applied [MPa]	Case 0 (Healthy)	Case 1	Case 2	Case 3	Compression Strength Limit of Tissue [MPa]
Femoral cartilage	0.38	under 1	1	1	1	5–20
	0.76	4.2	2.2	3.1	3.2	
Tibial cartilage	0.38	under 1	2.1	2.3	2.6	
	0.76	2.0	4.6	3.8	3.5	
UC	0.38	N/A	7.8	12.7	15.6	5–100
	0.76	N/A	25.3	22.9	18.8	
Femoral subchondral bone	0.38	5.3	5.6	9.7	11.6	64
	0.76	16.2	14.3	12.4	15.2	
Tibial subchondral bone	0.38	2.1	3.6	1.2	3.4	
	0.76	5.3	5.8	5.6	5.9	
Femoral trabecular bone	0.38	under 1	2.8	2.5	2.6	2–16
	0.76	3.4	5.2 *	3.6	5.8 *	

* Hight values determined in apex area of reconstruction; Case 1 (just porous Collagen implant); Case 2 (Collagen + BMC); Case 3 (Collagen + ASC).
